# Heterogeneous cellular effects of α-ketoglutarate esters

**DOI:** 10.18632/aging.102028

**Published:** 2019-06-11

**Authors:** Lorenzo Galluzzi

**Affiliations:** 1 Department of Radiation Oncology, Weill Cornell Medical College, New York, NY 10065, USA; 2 Sandra and Edward Meyer Cancer Center, New York, NY 10065, USA; 3 Department of Dermatology, Yale School of Medicine, New Haven, CT 06510, USA; 4 Université Paris Descartes, Paris, France

**Keywords:** acetyl-CoA, apoptosis, ATP, Krebs cycle, NAD ^+^, oxidative phosphorylation, starvation

Macroautophagy (herein referred to as autophagy) is an evolutionary old process whereby eukaryotic cells get rid of superfluous or potentially dangerous cytosolic material through lysosomal degradation [1]. Although components of the molecular machinery for autophagy can etiologically contribute to the demise of cells responding to perturbations of homeostasis, *bona fide* autophagic responses most often mediate cytoprotective effects [2]. Thus, eukaryotic cells generally rely on autophagy for adapting to a wide panel of potentially detrimental alterations of their microenvironment, including (but not limited to) nutritional and metabolic cues [3]. In particular, while nutrient-rich conditions potently repress autophagic responses as a consequence of AMP-activated protein kinase (AMPK) inhibition and mechanistic target of rapamycin (MTOR) complex 1 (MTORC1) hyperactivation, the deprivation of essential metabolites including some amino acids and (less to) glucose, drives autophagy downstream of AMPK activation and MTORC1 inhibition [2]. Thus, the overall nutritional status of a cell has a major impact on autophagy regulation [4].

Consistent with this notion, multiple metabolites that directly or indirectly impinge on ATP production by glycolysis or oxidative phosphorylation, including multiple intermediates of the tricarboxylic acid (TCA) cycle, have been shown to modulate autophagy *in vitro* and *in vivo* [4]. For instance, elevated cytosolic levels of acetyl coenzyme A (acetyl-CoA), which generally reflect good nutrient availability, are associated with autophagy suppression [5,6]. Conversely, cytosolic acetyl-CoA shortage, which generally indicates at least some degree of nutrient deprivation, is linked with autophagy activation [5,6]. Based on the same principle, high levels of α-ketoglutarate (an intermediate of the TCA cycle generated by the oxidative decarboxylation of isocitrate) are also expected to suppress autophagy, and multiple groups obtained preclinical data in support of this notion [5]. However, α-ketoglutarate has also been suggested to inhibit MTOR signaling and ATP production by oxidative phosphorylation, two conditions that would rather support autophagy activation [7]. 

One of the issues with the use of α-ketoglutarate for experimental purposes originates from the fact that the molecule is membrane impermeant. Thus, supplementing experimental systems with exogenous α-ketoglutarate fails to directly alter intracellular α-ketoglutarate levels. To circumvent this issue, α-ketoglutarate is often employed in one of its esterified variants, including dimethyl α-ketoglutarate (DMKG), trifluoromethylbenzyl α-ketoglutarate (TFMKG) and octyl α-ketoglutarate (OKG). These esters can indeed freely cross the plasma membrane to reach cytosolic esterases that release α-ketoglutarate and hence promote cellular accumulation. Recent data from the Kroemer laboratory reveal that all these esters inhibit autophagic responses driven by nutrient deprivation, but otherwise have heterogeneous effects on baseline autophagy, oxidative phosphorylation and cellular ATP content [8]. These findings may explain, at least in part, previous, apparently contradicting data on autophagy inhibition *versus* activation by α-ketoglutarate.

Baracco and collaborators demonstrated that human osteosarcoma U2OS cells treated with DMKG, TFMKG and OKG exhibit increased intracellular levels of α-ketoglutarate and decreased NAD^+^ levels (compatible with improved output from the TCA cycle), but virtually no other convergent metabolic alterations when the experiment was performed in complete culture medium. In nutrient-depleted conditions, a total of 24 intracellular metabolites were altered in a similar manner by DMKG, TFMKG and OKG, but the vast majority of changes were not shared by all three esters. Such semi-private alterations included the accumulation of acetyl-CoA (which was provoked by DMKG and TFMKG, but not OKG) and the depletion of ATP (which was caused by TFMKG and OKG, but not DMKG) [8]. From a functional perspective, all esters inhibited autophagic responses to nutrient deprivation, as assessed gold standard assays measuring the size of autophagic compartments and *bona fide* autophagic flux. Conversely, only TFMKG and OKG (but not DMKG) drove autophagic responses when U2OS cells were cultured in complete medium. Moreover, OKG was the only α-ketoglutarate ester that efficiently blocked oxidative phosphorylation, in a manner that did not depend on octanol (the other product of OKG de-esterification). Consistent with this activity, OKG (but not DMKG, TFMKG and octanol) mediated cytotoxic effects, both in U2OS cells and in yeast cells subjected to chronological aging experiments [8].

In summary, the only biological activity shared by α-ketoglutarate esters in this set of experiments was the inhibition of autophagy. Although the precise molecular mechanisms underlying the heterogeneity in the other effects of α-ketoglutarate esters remain to be elucidated, these data place emphasis on the existence of often underappreciated confounders even in simple experimental settings.
